# Motivation in Nursing: A Concept Analysis

**DOI:** 10.1002/nop2.70717

**Published:** 2026-07-28

**Authors:** Edward Obeng Amoah, Mopelola T. Adeola

**Affiliations:** ^1^ SDA Nursing and Midwifery Training College Kwadaso, Kumasi Ghana; ^2^ School of Nursing, Johnson Hall of Nursing Purdue University West Lafayette Indiana USA

**Keywords:** concept analysis, extrinsic motivation, intrinsic motivation, motivation, newly licensed registered nurses, nursing retention, nursing workforce, professional commitment, Walker and Avant, work motivation

## Abstract

**Aim:**

To clarify and report an analysis of the concept of motivation in nursing.

**Background:**

Motivation shapes nurses' career decisions, job satisfaction and long‐term retention. Persistent workforce challenges, particularly high turnover among newly licensed registered nurses, demand a clear, context‐specific understanding of nursing motivation. This concept analysis aimed to clarify motivation within nursing practice, identify its defining attributes, antecedents and consequences, and propose a nursing‐specific definition to guide workforce development, recruitment and retention strategies.

**Design:**

A concept analysis.

**Data Sources:**

A targeted literature review spanning nursing, psychology and organizational behaviour was conducted using PsycINFO, PubMed, CINAHL, Scopus and Google Scholar. Articles published between 2015 and 2026 were screened and selected to refine the conceptualization of motivation in nursing.

**Methods:**

Walker and Avant's method for analyzing concepts in eight steps was used.

**Results:**

Five defining attributes emerged: intrinsic drivers rooted in altruism and personal identification with the profession, extrinsic reinforcement from the work environment, goal‐directed professional actions, persistence and resilience in navigating challenges, and sustained commitment to both the profession and patient care. Motivation in nursing is a dynamic, multifaceted construct shaped by internal values, external conditions and fulfilment of basic psychological needs. It initiates, guides and maintains behaviours that support professional growth, high‐quality care and long‐term engagement.

**Conclusion:**

These findings underscore the need for practice environments that nurture intrinsic motivation while providing adequate extrinsic support through effective leadership, safe staffing, recognition, career pathways, wellness initiatives and mentorship, particularly for newly licensed registered nurses. The identified attributes and related elements offer a framework for designing motivation‐enhancing interventions and guiding future research in diverse nursing contexts.

**Implications for Nursing Practice:**

Nurse leaders and educators can apply these findings by implementing structured mentorship, recognition programmes and professional development pathways that strengthen intrinsic motivation while ensuring adequate staffing and organizational support, particularly during the transition to practice for newly licensed registered nurses.

**Patient or Public Contribution:**

No Patient or Public Contribution. This is a concept analysis based on a literature review; it does not involve primary data collection from patients or members of the public, and therefore, patient or public involvement was not applicable.

## 1 Introduction

Motivation plays an important role in the lives of healthcare professionals and influences decisions in various domains, including career choices, job satisfaction and retention among newly licensed registered nurses (NLRNs) (McCoach and Flake 2018; Ait Ali et al. 2024). Recognizing the importance of motivation in nursing is crucial for improving workplace environments, reducing turnover rates and enhancing patient care (American Nurses Association [ANA] 2023a; Mohammed et al. 2024). The shortage of nurses poses a considerable challenge to healthcare systems worldwide (ANA 2023b). Recent data indicate that maintaining adequate nursing staff remains an ongoing struggle, with burnout rates among nurses being persistently high (Omoregie and Yusuf 2025; Alansari et al. 2025).

According to the World Health Organization (WHO 2020), there is an estimated global deficit of 5.9 million nurses, emphasizing the urgent need for effective recruitment and retention strategies within the healthcare sector. To address these workforce challenges, it is critical to identify what motivates individuals to choose nursing as a career and to continue working in the field. Price and McGillis Hall (2014) and Halcomb et al. (2020) underscore that, given global nursing shortages and high turnover rates, particularly among newly licensed nurses, understanding what motivates people to enter, stay in, and excel in nursing is essential. The nursing profession has undergone substantial evolution over time, resulting in corresponding shifts in motivational factors that influence career choices and professional dedication (Guerrero et al. 2017; Duran et al. 2021). While motivation is often discussed in nursing literature, there is no universally accepted definition tailored to the nursing field. Motivation has been explored using theories such as self‐determination theory (Ryan and Deci 2000), Herzberg's Two‐Factor Theory (Herzberg et al. 1959), and various expectancy theories; however, these frameworks have not been combined to form a clear, unified view of motivation within nursing practice.

Previous concept analyses have explored motivation in various contexts, including health‐seeking behaviours, across different populations (Hooper and Quallich 2016; Poortaghi et al. 2015). Despite these efforts, no published concept analysis has applied Walker and Avant's framework specifically to define motivation within nursing practice and workforce development in the context of contemporary challenges such as NLRN turnover and burnout. Moreover, motivation in nursing is conceptually distinct from related constructs that are sometimes used interchangeably in the literature. Work engagement refers to a positive affective‐cognitive state characterized by vigour, dedication and absorption (Kohnen et al. 2023), and, while related to motivation, does not capture the initiating function or the altruistic orientation central to nursing motivation. Professional commitment describes an affective bond with and identification with the nursing profession (Guerrero et al. 2017) but is better understood as an outcome of sustained motivation rather than as its equivalent. Resilience reflects the capacity to recover from adversity (Mohammad et al. 2023), and intention to stay is a downstream behavioural intention, not a motivational driver. By delineating these boundaries, this concept analysis advances conceptual clarity and provides a nursing‐specific foundation that existing frameworks have not yet offered.

### 1.1 Aims

The purpose of this concept analysis is to clarify and define motivation as it relates specifically to nursing practice and workforce development. By examining nurses' unique experiences and perspectives regarding motivation in their professional roles, this analysis will provide healthcare leaders and stakeholders with vital information needed for the development of more effective strategies for the recruitment, retention and advancement of nurses.

## 2 Method

Walker and Avant's (2019) eight‐step framework guided the completion of this conceptual analysis. The specific steps taken were as follows: (a) select a concept, (b) determine the aim or purpose of the analysis, (c) identify all uses of the concept that can be discovered, (d) determine the defining attributes, (e) identify a model case, (f) identify borderline, related, contrary, invented and illegitimate cases, (g) identify antecedents and consequences and (h) define empirical referents (Walker and Avant 2019).

## 3 Data Sources

Given the concept under review (step one) and the purpose of the concept analysis (step two), step three of Walker and Avant's (2019) approach requires identifying the concept's definitions and uses. A literature review was conducted, which included the disciplines of nursing and psychology. PsycINFO, PubMed, CINAHL, Scopus and Google Scholar databases were searched to identify relevant articles for this concept analysis, and articles from 2015 to 2026 were included as the primary date range. Search terms used were ‘motivation in nursing’, ‘nurse motivation’, ‘intrinsic motivation’, ‘extrinsic motivation’, ‘work motivation’, ‘nursing retention’, ‘newly licensed registered nurses’, ‘professional commitment’, ‘nursing workforce’, ‘burnout nursing’, ‘concept analysis nursing’, and ‘self‐determination theory nursing’. These search terms were combined using Boolean operators (AND, OR). Articles were included if they were (a) peer‐reviewed journal articles or scholarly books; (b) published in English; (c) focused on motivation, work motivation, professional commitment, retention or related constructs in nursing, healthcare or organizational behaviour; and (d) published between 2015 and 2026. Articles were excluded if they were: (a) focused on patient motivation or motivation in non‐healthcare populations; and (b) non‐peer‐reviewed sources. Screening was conducted in two stages: titles and abstracts were first reviewed for relevance, followed by a full‐text review of eligible articles. The final search was executed in December 2025. Articles were selected if they directly contributed to conceptualizing, defining or measuring motivation in the context of nursing practice or healthcare work. Although the primary search was limited to 2015–2026, foundational theoretical and methodological works that predate this range were intentionally retained.

## 4 Results

### 4.1 Defining Motivation in Nursing

The literature lacks a single unified definition of the concept of motivation, despite its mention in numerous research articles and healthcare disciplines. General psychological definitions describe motivation as a process that initiates, guides and maintains goal‐oriented behaviour (Ryan and Deci 2000). McCoach and Flake (2018) defined motivation as the process by which goal‐directed behaviour is instigated and sustained. Pinder (2008) described work motivation as a set of energetic forces that originate both within and beyond an individual's being to initiate work‐related behaviour and to determine its form, direction, intensity and duration. The term ‘motivation’ can have different meanings for different professions and individuals, and this is also true for the concept of motivation in nursing. Within the context of clinical practice, motivation in nursing has been examined in relation to job performance, burnout and retention. Ait Ali et al. (2024) described career motivation as the primary factor propelling individuals to make choices and take action to accomplish both personal and organizational objectives. This definition highlights how motivation supports ongoing career commitment, which is a key factor in considering nursing's high turnover and burnout rates. While the broader literature uses related terms such as work motivation and career motivation, this analysis adopts ‘motivation in nursing’ as its unifying construct, encompassing the intrinsic and extrinsic forces specific to the nursing role; this term is used consistently throughout.

### 4.2 Critical Attributes/Defining Attributes

Throughout the literature review, several recurring themes emerged as critical attributes of nursing motivation. Critical attributes refer to characteristics that are consistently observed across multiple studies and sources, serving to distinguish the concept from other related concepts (Walker and Avant 2019). By identifying shared attributes, this concept analysis can clarify what truly defines motivation in the context of nursing practice.

The critical attributes of the concept of motivation in nursing as noted in the literature include: (a) intrinsic drivers, including altruistic orientation and personal connection to nursing (Guerrero et al. 2017; Mohammed et al. 2024; Ten Hoeve et al. 2017); (b) extrinsic reinforcement from workplace factors, including supportive environments, adequate resources and recognition (Alshammari and Alenezi 2023; Sutanto et al. 2023); (c) goal‐directed action towards professional objectives (McCoach and Flake 2018; Pinder 2008; Ryan and Deci 2017); (d) persistence and resilience in facing workplace challenges (Halcomb et al. 2020; Mohammad et al. 2023); and (e) commitment to the profession and patient care (Ait Ali et al. 2024; Mohammed et al. 2024).

Nurses encounter various professional and personal influences that shape their motivation to work (Mohammed et al. 2024). A significant portion of the literature highlights the importance of intrinsic factors such as altruism, the desire to help others and a personal connection to the nursing profession as central components of nursing motivation. These intrinsic drivers frequently stem from personal values, meaningful life experiences or exposure to the impact of health care on family members. The first critical attribute of nursing motivation reflects the foundational influence of these internal factors.

Extrinsic factors also play a key role in shaping nursing motivation, as demonstrated in more than half of the reviewed studies (Alshammari and Alenezi 2023). The workplace environment, which includes supportive colleagues and supervisors, access to adequate resources, appropriate staffing levels and professional recognition, has a substantial impact on nurses' motivation (Alshammari and Alenezi 2023; Sutanto et al. 2023). These external reinforcements can either strengthen or diminish nurses' intrinsic motivation, depending on whether such support is present or lacking (Ryan and Deci 2000; Silva and Menino 2022).

The third attribute of goal‐directed action consistently appears in the literature and aligns with general theories of motivation (McCoach and Flake 2018; Pinder 2008). Nurses exemplify motivation by striving for professional objectives, such as obtaining specialty certifications, pursuing advanced degrees, attaining leadership roles or striving for excellence in patient care delivery. Persistence and commitment were the fourth and fifth most critical attributes, respectively. Halcomb et al. (2020) and Mohammad et al. (2023) demonstrate that these qualities reflect the resilience nurses must maintain despite challenging work conditions, emotional stresses and physical demands inherent in the profession.

### 4.3 Definition

After considering the aforementioned attributes, motivation in nursing can be defined as a dynamic and multidimensional construct arising from both intrinsic and extrinsic factors that initiate, direct and sustain goal‐oriented behaviours towards professional development, quality patient care, and career persistence while being influenced by the satisfaction of basic psychological needs. Motivation emerges from personal values and altruistic orientation, is reinforced by supportive workplace environments, and manifests as a persistent commitment to patient care and professional development despite workplace challenges.

### 4.4 Antecedents

Antecedents are events that must precede a concept (Walker and Avant 2019). To be motivated in nursing, a person first decides to enter or stay in the profession and must have key values such as altruism and commitment to healthcare. Educational preparation and personal exposure to healthcare, such as through experiences, family or volunteering, typically precede the motivation to become a nurse. Finally, there must be awareness of nursing as a career option and an understanding of what the profession entails (Price and McGillis Hall 2014; Ten Hoeve et al. 2017; Ryan and Deci 2017). Table 1 below shows the conceptual framework that attempts to define the concept of motivation in nursing.

**TABLE 1:** Conceptual framework of motivation in nursing: Antecedents, defining attributes and the consequences of supported versus diminished motivation.

Stage in the framework	Key elements
Antecedents	Decision to enter or remain in nursing; core values of altruism and commitment to healthcare; educational preparation; personal exposure to healthcare (life experience, family or volunteering); awareness and understanding of nursing as a career
Defining attributes of motivation in nursing	Intrinsic drivers (altruistic orientation and personal connection to nursing); extrinsic reinforcement (supportive environment, adequate resources and recognition); goal‐directed professional action; persistence and resilience; commitment to the profession and patient care
Consequences of supported motivation (Positive Consequences)	Increased job satisfaction; enhanced patient care outcomes; lower turnover; greater engagement in professional development; stronger teamwork and collaboration; improved resilience against burnout
Consequences of diminished or unsupported motivation (Negative Consequences)	Burnout and emotional exhaustion; intention to leave the profession; diminished quality of care; higher rates of medication errors; weakened professional identity

*Note:* The table depicts the antecedents and defining attributes that shape motivation in nursing, along with the consequences of supported versus diminished motivation. Positive consequences (e.g., job satisfaction, lower turnover, professional growth and resilience) arise when motivation is sustained by both intrinsic and extrinsic factors, whereas negative consequences (e.g., burnout, higher turnover, diminished quality of care and weakened professional identity) reflect diminished or unsupported motivation. Motivational environments and outcomes influence one another bidirectionally over time.

### 4.5 Consequences

Consequences are defined as events or incidents that arise because of motivation in nursing (Walker and Avant 2019). The outcomes of motivation among nurses can manifest in both positive and negative forms, heavily influenced by the circumstances and degree of support provided within the work environment. When nurses experience high levels of motivation driven by intrinsic factors, such as commitment to patient care, and extrinsic factors, such as supportive work environments and adequate resources, the outcomes are generally beneficial. Positive consequences include increased job satisfaction, enhanced patient care outcomes, lower turnover rates, greater engagement in professional development, stronger teamwork and collaboration, and improved resilience against burnout (Mahmoud et al. 2020; Mohammed et al. 2024; Sutanto et al. 2023). In such settings, motivated nurses are more likely to attain fulfilment in their roles and remain committed to their profession.

In contrast, when workplace environments fail to nurture intrinsic motivation through factors such as inadequate staffing, lack of resources, poor leadership or insufficient recognition, motivation can decline. Alshammari and Alenezi (2023) and Halcomb et al. (2020) identify an increased risk of burnout and intention to leave the profession as key negative outcomes of diminished motivation. Vázquez‐Calatayud and Eseverri‐Azcoiti (2023) further associate reduced motivation with diminished patient care quality, higher rates of medication errors and a weakened sense of professional identity. These adverse effects are particularly pronounced among newly licensed registered nurses, who, despite entering the field with strong intrinsic motivation, may become disillusioned if their professional experiences do not align with their values and expectations.

### 4.6 Empirical Referents

Empirical referents are techniques used to measure or observe concepts. Several validated instruments have measured nursing motivation and related constructs. The Work Extrinsic and Intrinsic Motivation Scale (WEIMS) is an 18‐item scale that measures six types of motivation based on self‐determination theory, which includes intrinsic motivation, integrated regulation, identified regulation, introjected regulation, external regulation and amotivation (Tremblay et al. 2009).

The Multidimensional Work Motivation Scale (MWMS) is a 19‐item, self‐determination‐based instrument developed to measure work motivation across occupational settings, including healthcare. It assesses a full continuum of motivation through subscales for intrinsic motivation, identified regulation, introjected regulation, external regulation (material and social) and amotivation. The MWMS was validated using data from 3435 workers across seven languages and nine countries and demonstrates a consistent factor structure across samples. It shows predictable relationships with workplace antecedents and outcomes, including commitment, performance and turnover intentions, supporting its use in examining motivation within the nursing workforce (Gagné et al. 2015).

The Maslach Burnout Inventory (MBI) is used as an inverse measure of motivation in nursing. It assesses three domains: emotional exhaustion, depersonalization and reduced personal accomplishments. Higher MBI scores are typically associated with lower levels of motivation, as elevated burnout often indicates diminished engagement and drive among nurses (Ang et al. 2016).

### 4.7 Case Studies

This section comprises three case studies that clarify the concept of motivation in nursing. The first is a model that showcases all antecedents and attributes. The second case presented is a borderline case that contains some, but not all, of the attributes. The third and final case is a contrary case that does not include any defining attributes (Walker and Avant 2019). Related, invented and illegitimate cases were not constructed, consistent with Walker and Avant's (2019) guidance that additional cases are developed only when needed to clarify the concept's boundaries.

#### 4.7.1 Model Case

Sarah is a 28‐year‐old registered nurse who has been working in the intensive care unit for 3 years. She chose nursing because her grandmother received excellent care during a serious illness, and Sarah wanted to provide the same compassionate care to others. Sarah's intrinsic motivation stems from deeply caring about her patients' well‐being and finding meaning in easing their suffering during a critical illness. She voluntarily attends continuing education courses and recently enrolled in a critical care certification programme, demonstrating goal‐directed action towards professional development. Sarah works in a supportive environment in which her nurse manager recognizes excellent patient care, adequate staffing allows time for thorough assessments, and colleagues collaborate effectively as a team. These extrinsic factors reinforce Sarah's intrinsic motivation. When faced with challenging patient situations or emotionally difficult shifts, Sarah demonstrates resilience and persistence, reflecting on her experiences with her mentor and using them as learning opportunities. She remains committed to her patients and the nursing profession, even during stressful periods.

In this model, Sarah exemplifies all five critical motivational attributes in nursing. She demonstrates intrinsic drivers through altruistic orientation and personal connections with nursing. She benefits from extrinsic reinforcement in a supportive workplace. She engages in goal‐directed actions through professional development. She demonstrates persistence and resilience when facing workplace challenges. Finally, she maintains her commitment to the profession and patient care despite the demands of work.

#### 4.7.2 Borderline Case

Michael, a newly licensed registered nurse, is goal‐oriented, consistently seeks promotions and meets the performance metrics. However, his focus is on career advancement rather than on direct patient care. His motivation is highly dependent on the recognition and monetary incentives provided by the supervisor. When faced with workplace challenges, Michael considers alternative career options to developing resilience strategies to manage stress. Although he demonstrates some motivational attributes, his minimal focus on resilience results in a weaker long‐term commitment to nursing. This case highlights an imbalance in motivation, where extrinsic incentives drive performance, but intrinsic passion and resilience are underdeveloped.

#### 4.7.3 Contrary Case

Jennifer is a 25‐year‐old registered nurse who graduated 6 months ago and accepted a position in the emergency department. She chose nursing because her parents encouraged her to pursue a healthcare career, even though she had no particular interest in patient care. Jennifer finds work emotionally exhausting and physically demanding. She frequently calls in sick, arrives late to shifts and performs the minimum required work to avoid being fired. Jennifer does not seek learning opportunities, avoids complex patient situations and spends breaks complaining about her job with colleagues. Despite working in a supportive department with excellent mentorship and reasonable staffing, Jennifer remains disillusioned. She makes no effort to improve her skills, does not pursue specialty certification, and openly discusses her plans to leave nursing for a completely different career within the next year.

In contrast, Jennifer does not exhibit any of the defining attributes of motivation in nursing. She lacks intrinsic drivers such as altruistic orientation or personal connections to the profession. Although extrinsic reinforcement is available through her supportive workplace, it does not affect her engagement. She demonstrates no goal‐directed actions towards professional development. She shows no persistence or resilience when faced with normal workplace challenges. Finally, she has no commitment to either patient care or nursing. Jennifer is an example of amotivation in nursing.

## 5 Discussion

This concept analysis clarifies motivation in nursing as a multidimensional, dynamic psychological state fundamentally shaped by the interplay of intrinsic and extrinsic factors, satisfaction with psychological needs, goal‐directed energy and contextual influences (McCoach and Flake 2018; Pinder 2008; Alshammari and Alenezi 2023). Understanding this concept has significant implications for nursing education, practice and healthcare organizations. The analysis identified multiple defining attributes of motivation in nursing, including intrinsic drivers rooted in altruistic orientation, extrinsic reinforcement, goal‐directed action, persistence and resilience, and commitment to the profession and patient care. Sutanto et al. (2023) and Ryan and Deci (2017) highlight that these attributes reflect the complex and dynamic nature of motivation in the nursing context, highlighting both internal psychological processes and external environmental influences that shape nurses' professional involvement.

The five defining attributes identified in this analysis align with and extend several major theoretical frameworks, deepening the theoretical grounding of nursing motivation beyond prior literature. Self‐Determination Theory (SDT; Ryan and Deci 2000, 2017) provides the most robust theoretical anchor: the attributes of intrinsic drivers, extrinsic reinforcement and goal‐directed action map directly onto SDT's three basic psychological needs of autonomy, competence and relatedness. When these needs are satisfied in the clinical environment, SDT predicts the internalization of external regulation into identified and integrated motivation, which is precisely what the persistence and commitment attributes reflect. Kohnen et al. (2023) found that intrinsic motivation mediates the relationship between the perceived clinical work environment and work engagement, suggesting that the motivational attributes identified here operate through mechanisms consistent with SDT. Professional identity formation theory further contextualizes the intrinsic driver attribute: nurses' altruistic orientation and personal connection to nursing are not merely preferences but reflect an evolving professional self‐concept shaped through education, socialization and practice (Ten Hoeve et al. 2017; Guerrero et al. 2017).

This has direct implications for nursing education curricula; those that cultivate professional identity and values‐based practice are likely to strengthen the antecedents of motivation before nurses enter the workforce. Work engagement theory (Bakker and Leiter 2010, as applied by Kohnen et al. 2023) complements this analysis by distinguishing motivated states from engaged states: motivation initiates and directs behaviour, while engagement reflects its sustained experiential quality. Finally, the American Association of Critical‐Care Nurses' Healthy Work Environment (HWE) standards (AACN 2016), including skilled communication, true collaboration, effective decision‐making, appropriate staffing, meaningful recognition and authentic leadership, align closely with the extrinsic reinforcement attribute identified here. Healthcare institutions that implement HWE standards create the structural conditions most likely to sustain motivation across all five attributes.

Healthcare leaders, educators and policymakers can play pivotal roles in enhancing nurses' motivation at all career stages. Given the critical attributes identified as intrinsic drivers, extrinsic reinforcement, goal‐directed action, persistence and commitment, the most important implication for healthcare organizations is the creation of environments that support both intrinsic and extrinsic motivation (Bailey 2019; Bandhu et al. 2024). Supporting nurses' intrinsic motivation requires acknowledging the altruistic values and personal connections that draw them to nursing and ensuring that these values are not eroded by workplace conditions. Ryan and Deci (2017) and Silva and Menino (2022) argue that providing extrinsic reinforcement through supportive leadership, adequate resources, appropriate staffing and professional recognition can strengthen, rather than undermine, intrinsic motivation.

Duran et al. (2021) demonstrated how the COVID‐19 pandemic revealed that altruistic motivation sustains nurses' commitment even under extraordinary stress, suggesting that recruitment strategies should nurture this core nursing identity while providing adequate organizational support. Khomkham and Kaewmanee (2024) and Zakaria et al. (2024) found that clear professional goals and structured career development pathways significantly enhance nurses' motivation and persistence. The goal‐directed action attribute suggests that organizations should provide clear pathways for professional development, including continuing education opportunities, specialty certifications, advanced practice roles and leadership positions. Making these opportunities visible and accessible demonstrates organizational investment in nurses' career advancement. Healthcare leaders should create structures that support nurses' professional goals while aligning them with organizational objectives (Mohammed et al. 2024).

Persistence, resilience and commitment highlight the need for ongoing support. Mentorship programmes, peer support groups, wellness initiatives and mental health resources can help nurses maintain their motivation, despite the emotional and physical demands of the profession. This is particularly important for newly licensed registered nurses who may experience reality shock when workplace conditions do not meet their expectations (Vázquez‐Calatayud and Eseverri‐Azcoiti 2023). Organizations should implement structured transition programmes that help new nurses develop resilience while maintaining the intrinsic motivation that brings them to nursing in the first place.

Understanding the antecedents of nursing motivation, personal values, educational preparation, healthcare exposure and career awareness has implications for recruitment and education in nursing. Nursing programmes should help students develop a clear understanding of professional realities while nurturing altruistic values that support long‐term motivation. Healthcare organizations should participate in outreach efforts that expose young people to nursing careers, particularly in underserved communities where healthcare workforce shortages are the most severe (Price and McGillis Hall 2014).

The consequences of nursing motivation, both positive and negative, underscore the importance of organizational investment in creating a motivating environment. Mohammed et al. (2024), Mahmoud et al. (2020) and Sutanto et al. (2023) demonstrate that the positive consequences of enhanced motivation include improved patient outcomes, increased nurse retention, stronger professional development and greater organizational effectiveness. Alshammari and Alenezi (2023) and Halcomb et al. (2020) identify diminished motivation as a contributor to burnout, turnover, reduced quality of care and loss of experienced nurses from the workforce. These consequences represent not only individual and organizational costs but also broader costs to the healthcare system and society.

The conceptual framework developed in this analysis carries specific implications across four domains. In nursing education, the identified antecedents, personal values, healthcare exposure, professional awareness and educational preparation suggest that motivation development begins before clinical practice. Nursing curricula should incorporate early values clarification, professional socialization activities and realistic previews of practice to cultivate intrinsic motivation and professional identity before workforce entry. Simulation‐based learning and mentored clinical experiences during education can strengthen goal‐directed action and resilience as motivational attributes before NLRNs encounter the demands of acute practice. In workforce policy, the antecedent‐attribute‐consequence framework developed here provides an evidence‐based structure for evaluating nursing workforce interventions. Policymakers should prioritize safe staffing standards, compensation equity and protected time for professional development as structural extrinsic reinforcers. In intervention development, the five defining attributes offer specific targets: programmes aimed at intrinsic drivers may use narrative reflection or values‐based supervision; those targeting extrinsic reinforcement may focus on leadership development or Magnet designation criteria; and those targeting resilience may incorporate structured peer support or transition‐to‐practice programmes. In future measurement, the empirical referents identified here (the WEIMS, MWMS and MBI) provide a starting point, but the field would benefit from a nursing‐specific motivation measure that operationalizes all five attributes simultaneously and is validated across diverse clinical and cultural contexts.

### 5.1 Implications for Nursing Practice

The findings of this concept analysis have direct application at the point of care, where nurse leaders and practicing nurses can use the identified attributes to design and evaluate motivation‐focused strategies. Because intrinsic drivers, extrinsic reinforcement, goal‐directed action, persistence and commitment operate together, unit‐level interventions are most effective when they address more than one attribute simultaneously. Nurse managers can strengthen intrinsic motivation by creating opportunities for nurses to reconnect with the altruistic and relational aspects of their work, such as structured reflection, patient‐outcome storytelling and recognition practices that highlight the meaning of care rather than only productivity metrics. Pairing these efforts with tangible extrinsic supports, including adequate staffing ratios, fair scheduling and visible career advancement pathways, reinforces rather than substitutes for intrinsic commitment (Ryan and Deci 2017; Silva and Menino 2022).

For newly licensed registered nurses, whose motivation is most vulnerable during the transition to practice, unit‐based mentorship and structured preceptorship programmes should explicitly target persistence and resilience, helping new nurses reframe early clinical setbacks as expected elements of professional growth rather than signals to leave the profession (Vázquez‐Calatayud and Eseverri‐Azcoiti 2023). Charge nurses and preceptors are well positioned to model goal‐directed behaviour by helping orientees set and track incremental clinical competencies, linking day‐to‐day tasks to longer‐term professional goals. Nursing practice councils and shared governance structures offer a practical mechanism for translating the attributes identified here into unit‐specific action plans, ensuring that motivation‐enhancing strategies are grounded in the realities of frontline practice rather than imposed solely as top‐down policy.

### 5.2 Limitations

This concept analysis offers a comprehensive definition of motivation in the nursing context. As with any literature review or concept analysis, this study has both strengths and weaknesses. One weakness is that some uses of the concept may have been overlooked in the reviewed literature. Another weakness is the possibility that certain nursing populations or practice settings may be under‐represented in the literature. The discussion also has limited generalizability outside the contexts represented in the literature, which predominantly include hospital‐based nursing in developed countries.

Despite these weaknesses, the concept analysis has several strengths. The analysis captured the attributes of motivation in nursing by including research from multiple disciplines, including nursing, psychology and organizational behaviour. The analysis provides insights for healthcare leaders, educators and policymakers into the factors that influence nursing motivation and strategies to enhance it. The identified attributes, antecedents and consequences provide a foundation for developing interventions to support nursing motivation and for future research to test this concept.

## 6 Conclusion

The global nursing shortage and high turnover rates, particularly among new nurses, highlight the importance of understanding and supporting nursing motivation. Nurses face unique challenges that influence their motivation through internal and external factors. Motivation is associated with job satisfaction, professional development, patient care quality and organizational stability.

Healthcare leaders and educators should consider fostering environments that support intrinsic motivation and provide clear paths for career growth, resources, leadership and recognition. Such efforts may be important for retaining newly licensed nurses and reducing early attrition. The attributes and relationships clarified in this analysis offer a basis for developing and testing evidence‐based strategies that could support job satisfaction, retention, patient outcomes and strengthen the nursing workforce.

## 7 Recommendations

Future research should test motivation‐enhancing interventions derived from this concept analysis, examine motivation factors in nurses, explore motivational dynamics in diverse nursing contexts beyond acute care, investigate cultural variations in nursing motivation, and develop context‐specific measures that capture the nuances of motivation in nursing. Nursing educators, leaders and policymakers must recognize that nurse motivation is not an individual responsibility, but rather a collective imperative that requires supportive educational environments, healthy workplaces, adequate compensation and systemic respect for nursing's essential contributions to healthcare.

## Author Contributions

Conceptualization, E.O.A. and M.T.A.; methodology, E.O.A.; formal analysis, E.O.A.; writing/original draft preparation, E.O.A.; writing – review and editing, E.O.A. and M.T.A.; supervision, M.T.A. All authors have read and agreed to the published version of the manuscript.

## Funding

The authors have nothing to report.

## Conflicts of Interest

The authors declare no conflicts of interest.

## Data Availability

Data sharing does not apply to this article, as no new data were created or analysed in this concept analysis.
